# Cerebrospinal Fluid Biomarkers for Kii Amyotrophic Lateral Sclerosis/Parkinsonism-Dementia Complex

**DOI:** 10.1155/2013/679089

**Published:** 2013-03-27

**Authors:** Yui Nakayama, Satoru Morimoto, Misao Yoneda, Shigeki Kuzuhara, Yasumasa Kokubo

**Affiliations:** ^1^Medical Department, Mie University School of Medicine, 2-174 Edobashi, Tsu, Mie 514-8507, Japan; ^2^Department of Neurology, Tokyo Metropolitan Geriatric Hospital, 35-2 Sakaemachi, Itabashi-ku, Tokyo 173-0015, Japan; ^3^Department of Medical Welfare, Suzuka University of Medical Science, 1001-1 Kishioka-cho, Suzuka City, Mie 510-0293, Japan; ^4^Department of Neurology, Mie University School of Medicine, 2-174 Edobashi, Tsu, Mie 514-8507, Japan

## Abstract

*Objective*. Amyotrophic lateral sclerosis/parkinsonism-dementia complex is classified as one of the tauopathies. *Methods*. The total tau, phosphorylated tau, and amyloid *β*42 levels were assayed in cerebrospinal fluid from patients with Kii amyotrophic lateral sclerosis/parkinsonism-dementia complex (*n* = 12), Alzheimer's disease (*n* = 9), Parkinson's disease (*n* = 9), amyotrophic lateral sclerosis (*n* = 11), and controls (*n* = 5) using specific enzyme-linked immunosorbent assay methods. *Results*. Total tau and phosphorylated tau did not increase and amyloid *β*42 was relatively reduced in Kii amyotrophic lateral sclerosis/parkinsonism-dementia complex. Relatively reduced amyloid *β*42 might discriminate Kii amyotrophic lateral sclerosis/parkinsonism-dementia complex from amyotrophic lateral sclerosis and Parkinson's disease, and the ratios of phosphorylated-tau to amyloid *β*42 could discriminate Kii amyotrophic lateral sclerosis/parkinsonism-dementia complex from Alzheimer's disease. *Conclusions*. Cerebrospinal fluid analysis may be useful to differentiate amyotrophic lateral sclerosis/parkinsonism-dementia complex from Alzheimer's disease, amyotrophic lateral sclerosis, and Parkinson's disease.

## 1. Introduction

Amyotrophic lateral sclerosis/parkinsonism-dementia complex (ALS/PDC) is a rare disorder endemic to Guam Island and the Kii Peninsula of Japan. It shows a unique combination of parkinsonism, amyotrophy, and dementia [1], and the form of dementia, which shows a phenotype similar to Alzheimer's disease (AD), is becoming predominant in the Kii Peninsula.

Although Kii ALS/PDC shows several unique clinical features, including severe atrophy of the frontal and temporal lobes on magnetic resonance imaging (MRI), decreased cerebral blood flow in the frontal and temporal lobes on single-photon emission computed tomography (SPECT) [2], pigmentary retinopathy [3], and decreased cardiac ^123^I-meta-iodobenzylguanidine uptake [4], a postmortem examination is required for a definitive diagnosis. Since biomarkers for ALS/PDC have not yet been identified, we analyzed cerebrospinal fluid (CSF) biomarkers for Kii ALS/PDC to discriminate it from other neurodegenerative disorders.

## 2. Material and Methods

We collected CSF samples from 12 patients with Kii ALS/PDC (6 men, 6 women, mean age 67.9 ± 3.7 years, mean illness duration 5.63 years), nine patients with AD (2 men, 7 women, mean age 61.1 ± 8.7 years, mean illness duration 1.92 years), 11 patients with ALS (8 men, 3 women, mean age 60.6 ± 12.6 years, mean illness duration 1.1 years), nine patients with Parkinson's disease (PD; 7 men, 2 women, mean age 71.3 ± 2.2 years, mean illness duration 4.42 years), and five disease control patients (C; 4 men, 1 woman, mean age 36.2 ± 20.3 years). All of the patients with Kii ALS/PDC were natives of Hohara village, which is an area of high ALS/PDC prevalence on the Kii Peninsula. We collected CSF samples over 10 years; therefore, the period between CSF collection and analysis was not standardized. The diagnosis of Kii ALS was made according to the Airlie House criteria, since the clinical symptoms of Kii ALS are essentially the same as those of classical ALS. The diagnosis of Kii PDC was made by a unique combination of levodopa-unresponsive parkinsonism and dementia, which are frequently accompanied by amyotrophy of the extremities and/or pyramidal tract signs. Mini-mental state examination (MMSE) was used for the evaluation of dementia and cut-off point was 23 (data not shown). The frontal lobes and/or temporal lobes of ALS/PDC patients showed atrophy on MRI and/or a decrease of cerebral blood flow on SPECT. CSF samples were immediately centrifuged at 1000 ×g for 15 min and stored at −80°C with polypropylene tube. The total tau (t-tau), phosphorylated tau (p-tau), and amyloid beta (A*β*) concentrations were measured with an enzyme-linked immunosorbent assay (ELISA) kit using a monoclonal antibody specific for t-tau, p-tau, and A*β*1–42 (INNOTEST hTAU Ag, phosphor tau(181P), and *β*-amyloid (1–42), Innogenetics, Ghent, Belgium). ELISA assays were carried out using several samples from each group on the same plate in a randomized manner and were repeated using randomized samples in the same manner in plural times. A factorial ANOVA was performed with CSF-t-tau, CSF-p-tau, and CSF-A*β*42, as dependent variables, with the diagnostic category (AD, ALS, C, Kii ALS/PDC, and PD) using JMP 9.0. All data were expressed as means ± SD. A *P* value less than 0.05 was considered statistically significant. The Ethics Committee of Mie University Graduate School of Medicine approved this study and the “Declaration of Helsinki” was followed. Informed consent was obtained from the patients or their families.

## 3. Results

**Figure 1:**
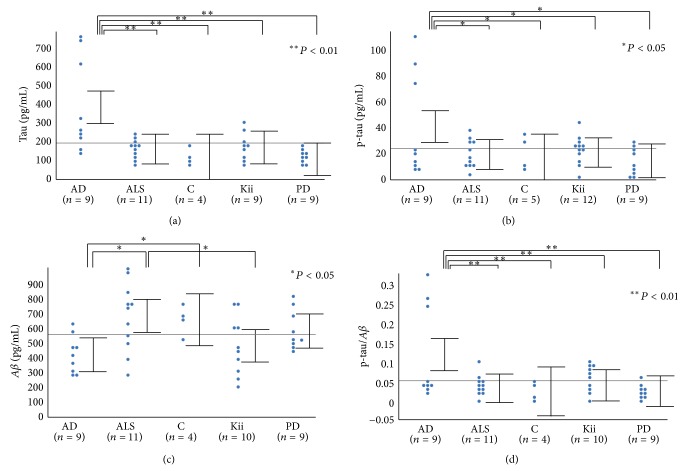
Analysis of CSF biomarkers. (a) Total tau, (b) phosphorylated tau, (c) beta-amyloid peptide (1–42), (d) the ratio of p-tau to A*β*42. AD: Alzheimer's disease, ALS: amyotrophic lateral sclerosis, C: disease control, Kii: Kii amyotrophic lateral sclerosis/parkinsonism-dementia complex, and PD: Parkinson's disease.

CSF-A*β*42, CSF-t-tau, and CSF-p-tau were compared between AD, ALS, C, Kii ALS/PDC, and PD. The concentrations of CSF-t-tau and CSF-p-tau were significantly higher in AD (t-tau; 378.0 ± 41.76 pg/mL; *P* < 0.001, p-tau; 42.4 ± 6.78 pg/mL; *P* < 0.028) than in the other groups. However, the concentrations of CSF-t-tau and CSF-p-tau did not differ significantly between Kii ALS/PDC, ALS, C, and PD (Figures 1(a) and 1(b)). The concentration of CSF-A*β*42 was significantly reduced in AD (402.2 ± 56.6 pg/mL; *P* < 0.03) compared to ALS and C and relatively reduced in Kii ALS/PDC (465.4 ± 53.69 pg/mL; *P* < 0.018) compared to ALS. Most of the ALS/PDC patients had CSF concentration values that fell below the cutoff based on C (Figure 1(c)). The ratios of CSF-p-tau to CSF-A*β*42 were significantly increased in AD (0.125 ± 0.02) compared with Kii ALS/PDC (0.043 ± 0.02; *P* < 0.008), ALS (0.035 ± 0.019; *P* < 0.003), PD (0.025 ± 0.02; *P* < 0.002), and C (0.027 ± 0.09; *P* < 0.014) (Figure 1(d)). The concentrations of CSF-t-tau, CSF-p-tau, or CSF-A*β*42 were not related to the clinical parameters (age, sex, illness duration, or dementia) in the Kii ALS/PDC patients. The number of C samples was small and the average age of control patients was low.

Generally, CSF tau level gradually increase according to age and CSF A*β* is not affected by age [5]. CSF tau level of C samples was relatively low, but it was not significant.

Thus, the CSF values of C were not comparable to those of other groups. Nevertheless, the optimal cut-off values that discriminate C from AD were similar to those in previous reports [6, 7] in which a larger number of control samples were analyzed. CSF tau level of Kii ALS/PDC samples did not increase, although the average age of Kii ALS/PDC group was older than that of AD group.

## 4. Discussion

The present study showed that CSF-t-tau and CSF-p-tau concentrations from patients with Kii ALS/PDC were not increased compared to those in the other disease groups, and A*β*42 concentration in the CSF was relatively decreased. The ratio of CSF-p-tau to CSF-A*β*42 segregates Kii ALS/PDC from AD. Because ALS/PDC is associated with tau pathology in the absence of amyloid plaques, the expectation was that ALS/PDC patients would not show the Alzheimer's disease (AD) profile of decreased A*β*42 but might show increased t-tau and/or p-tau in the CSF.

In general, decreased CSF-A*β*42 indicates plaque pathology, and increased CSF-t-tau and CSF-p-tau indicate axonal degeneration and tangle pathology, respectively [8]. Recently, the average age of onset of Kii ALS/PDC is increasing and A*β* deposition is conspicuous in autopsied patients. Therefore, decreased CSF-A*β*42 may reflect A*β* pathology in the most recent patients. We analyzed the precise tau isoform of over 10 patients with autopsy-proven ALS/PDC recently and identified a 3R + 4R type, 4R > 3R type, and a 4R predominant type. The glial tau pathology is particularly related to the 4R isoform, and we consider Kii ALS/PDC to be a 4R-dominant tauopathy (unpublished data). Noguchi et al. examined the concentrations of CSF-t-tau, CSF-p-tau, and CSF-A*β*42 in patients with progressive supranuclear palsy (PSP) and corticobasal degeneration (CBD); the concentrations of CSF-t-tau and CSF-p-tau did not significantly differ between PSP, CBD, and controls, and the concentration of CSF-A*β*42 was significantly lower in PSP and CBD than in controls. The authors speculated that the absence of an increase of CSF-t-tau and CSF-p-tau concentrations might reflect 4R tau predominance and a reduction of CSF-A*β*42 might suggest deposition or mismetabolism of A*β* [6]. Taken together, CSF biomarkers of Kii ALS/PDC might have similar properties to those of 4R tauopathy, PSP, and CBD; however the relationship between tau isoform and CSF tau level remains to be resolved.

Finally, the present findings, in which CSF-t-tau and CSF-p-tau concentrations were not increased and CSF-A*β*42 concentration was relatively decreased, suggest that CSF analysis may be useful to differentiate ALS/PDC from AD, ALS, and PD. Nevertheless there is a major limitation of the interpretation of the data. The size of each group is small, the age of the control group is much younger, and there were two populations in the AD group regarding the levels tau, p-tau, and A*β*/p-tau. Further study using groups with larger size of subjects is needed to confirm the proposed utility of the CSF biomarkers.

## Abbreviations

ALS/PDC: Amyotrophic lateral sclerosis/parkinsonism-dementia complex
CSF: Cerebrospinal fluid
NFTs: Neurofibrillary tangles
t-tau: Total tau
p-tau: Phosphorylated tau
AD: Alzheimer's disease
PD: Parkinson's disease
MRI: Magnetic resonance imaging
SPECT: Single-photon emission computed tomography
C: Control patients
ELISA: Enzyme-linked immunosorbent assay
3R: 3 repeat
4R: 4 repeat
PSP: Progressive supranuclear palsy
CBD: Corticobasal degeneration.
